# Nomogram to predict periprosthetic joint infection after total hip arthroplasty using laboratory tests

**DOI:** 10.1186/s10195-025-00833-2

**Published:** 2025-03-19

**Authors:** Junzhe Lang, Zetao Dong, Boyuan Shi, Dongdong Wang, Jiandong Yuan, Lei Chen, Jianqing Gao, Anan Sun, Jiyue Huang, Zhiqiang Xue

**Affiliations:** 1 Department of Orthopaedics, The First Affiliated Hospital of Wenzhou Medical University, Wenzhou, 325035 Zhejiang Province China; 2 Department of Clinical Medicine, Wenzhou Medical University, Wenzhou, 325035 Zhejiang Province China; 3 Operating Room, The First Affiliated Hospital of Wenzhou Medical University, Wenzhou, 325035 Zhejiang Province China; 4 Department of Orthopaedics, Guangzhou Red Cross Hospital of Jinan University, Guangzhou, 510220 Guangdong Province China; 5 Department of Orthopedics, The 900th Hospital of the People’s Liberation Army Joint Service Support Force, Fuzhou, 350000 Fujian Province China

**Keywords:** Total hip arthroplasty, Periprosthetic joint infection, Laboratory tests, Risk factors, Nomogram

## Abstract

**Background:**

Periprosthetic joint infection (PJI) is a catastrophic complication after joint arthroplasty. This study aimed to analyze the relationship between laboratory tests and PJI and establish a nomogram for predicting risks of PJI after total hip arthroplasty (THA).

**Materials and methods:**

The clinical data of patients who underwent THA from January 2015 to December 2020 were retrospectively analyzed. Demographic and relevant clinical information of patients was collected; independent risk factors associated with PJI were determined by univariate and multivariate logistic regression analysis, and receiver operating characteristics (ROC) were drawn to analyze the specificity and sensitivity of each risk factor. Risk factors are included in the nomogram. Calibration curve and decision curve analysis were used to evaluate the predictive accuracy and discriminability of the model.

**Results:**

A total of 589 patients were enrolled in the study, of whom 87 were eventually diagnosed with PJI. Multivariate logistic regression analysis showed that serum C-reactive protein, erythrocyte sedimentation rate, polymorphonuclear neutrophils, D-dimer, and platelet count were independent risk factors for PJI after THA. The ROC curve analysis model of multivariate combined diagnosis had good diagnostic value, sensitivity was 77.01%, and specificity was 75.51%. The calibration curve shows good agreement between the prediction of the line graph and the actual observed results. The decision curve shows that the nomogram has a net clinical benefit.

**Conclusions:**

The changes in serum C-reactive protein, erythrocyte sedimentation rate, polymorphonuclear neutrophils, D-dimer, and platelet count are related to the occurrence of PJI after hip arthroplasty. The nomogram prediction model established in this study is promising for the screening of PJI after hip arthroplasty.

*Level of evidence*: Level III evidence. Non-randomized controlled cohort/follow-up study.

## Introduction

Periprosthetic joint infection (PJI) is one of the primary reasons for revisions after total knee arthroplasty (TKA) and total hip arthroplasty (THA) and is associated with high morbidity rates and substantial economic burdens [1, 2]. Diagnosing PJI, particularly of the hip, is challenging due to the lack of a single definitive test [3]. The diagnostic process requires an integrated evaluation of clinical symptoms, patient history, auxiliary examination results, and intraoperative findings [4]. The complexity of the hip anatomy often impedes early detection of PJI, thereby delaying interventions and potentially exacerbating patient outcomes.

Recent studies highlight the potential of serological markers such as white blood cell count (WBC), erythrocyte sedimentation rate (ESR), and C-reactive protein (CRP) in diagnosing PJI [5]. The diagnostic criteria for PJI are based on updated criteria of the Musculoskeletal Infection Society (MSIS) [3]; the criteria for diagnosing PJI require meeting at least one primary diagnostic criterion or four minor criteria, with serological results playing a significant role. However, diagnosis remains comprehensive, involving both serological markers and clinical signs such as sinus tracts and pus. For patients with a high clinical suspicion of PJI, performing hip joint aspiration and culture and synovial fluid analysis is generally recommended. Still, joint puncture is an invasive operation that may cause iatrogenic infection and other risks [6, 7]. Variability in aspiration techniques and laboratory analyses can affect results. Therefore, quickly screening potential PJI patients through simple hematological indicators is crucial.

There is a gap in research on the combined use of various hematological indicators for evaluating PJI risk post-THA. Developing a visual scoring system based on serum tests could enable rapid assessment of potential PJI in patients with THA, prompting further examinations and improving prognosis. This study aims to identify and validate PJI risk factors using the MSIS criteria and determine optimal thresholds. We intend to create a nomogram for quick screening of potential PJI after THA, facilitating early diagnosis and management, which could enhance patient care and reduce healthcare costs.

## Materials and methods

This retrospective study, approved by the Ethics Committee of the First Affiliated Hospital of Wenzhou Medical University, analyzed patients who underwent THA between January 2015 and December 2020. Patients were followed up with at 3-, 6-, and 12-months postsurgery to assess wound status and limb function recovery. A comprehensive hematological examination was typically recommended at 3 months after discharge and recorded in the system. The study was based on this independent database.

Inclusion criteria were (1) an age ≥ 18 years, (2) complete pre- and postoperative medical records, and (3) continuous follow-up for at least 1 year. Exclusion criteria were (1) potential infectious diseases (respiratory, urinary, gastrointestinal, or skin/soft tissue) based on case records, admission examinations, physical examinations, and laboratory tests; (2) conditions that could affect serological indicators, including malignancies, severe liver dysfunction, coagulation disorders, and systemic inflammatory diseases (“Diseases including rheumatoid arthritis, systemic lupus erythematosus, psoriatic arthritis, ankylosing spondylitis, Sjogren’s syndrome, inflammatory bowel disease, systemic sclerosis, and autoimmune liver disease.”); (3) a diagnosis of aseptic loosening for noninfectious reasons (periprosthetic fractures, implant wear, instability, misalignment, or unexplained pain); and (4) history of joint revision arthroplasty.

A total of 589 patient records post-THA were included in this study. These records, encompassing hospitalization, readmission, rehabilitation, and outpatient services, were thoroughly reviewed to determine outcomes. Patients’ baseline information, such as age, sex, body mass index (BMI), side of surgery (left/right), smoking and drinking history, and comorbidities, was obtained from their initial hospital admission electronic medical records following THA. Some patients had their initial THA surgery elsewhere, and data for these cases were obtained from readmission and outpatient records. Collected data included laboratory tests such as complete blood count, biochemistry, coagulation function, and serum markers.

Laboratory data for patients with PJI were collected as part of routine presurgical examinations. Blood samples for fibrinogen, D-dimer, and other markers were taken on the morning after admission and then promptly sent to the medical laboratory center for testing. The laboratory data for patients No-PJI patients were primarily obtained from outpatient follow-up examinations. Through the database, we collected detailed hematological tests for patients, including liver function, kidney function, coagulation function, D-dimer, ESR, and CRP.

### Statistical analysis

Continuous variables were expressed as mean ± standard deviation (SD) or median (range) and compared using an independent Student *t*-test or Mann–Whitney test. Qualitative data are described in numbers and percentages and compared using chi-squared or Fisher exact tests where appropriate. *P* < 0.05 variables were substituted into multivariate logistics regression to identify independent risk factors, and LR (Likelihood Ratio) was used for variable selection. When the Youden index (sensitivity + specificity − 1) was the largest, the sensitivity and specificity of each variable for the diagnosis of PJI were found through ROC curve analysis, and the cutoff value was determined.

A predictive model was established, and a nomogram was created to calculate the total score of each risk factor as a risk score, providing a visual system for predicting PJI. The Hosmer–Lemeshow (H–L) goodness-of-fit test and calibration curve were used to evaluate the model’s fit. The area under the ROC and the optimal cutoff values were calculated to assess the model’s discriminative ability. Decision curve analysis (DCA) was used to evaluate the clinical validity and net benefit of the nomogram.

Statistical analysis was performed using SPSS (version 18.0; SPSS Company, Chicago, IL, USA) and R software version 3.22, and ROC curves were constructed by Med-Calc 20.0. Statistical significance was set as *P* < 0.05.

## Result

A total of 589 patients post-THA were included, of which 87 were PJI patients. The demographic characteristics and laboratory and clinical data of the PJI and No-PJI groups are summarized in Table 1. In the univariate analysis, potential risk factors for PJI after THA included age, BMI, CRP, ESR, WBC, platelet count (PC), D-dimer, and diabetes (*P* < 0.05). After entering each potential risk variable from Table 1 into a univariate logistics analysis, those with *P* < 0.05 were selected as candidate predictive variables to be included in the multivariate logistics. Ultimately, five independent risk factors were established: CRP (odds ratio [OR], 5.03; 95% confidence interval [CI] 2.74–9.12, *P* < 0.001), ESR (OR, 3.65; 95% CI 1.93–6.87, *P* < 0.001), PC (OR, 2.35; 95% CI 1.17–4.73, *P* = 0.017), D-dimer (OR, 3.55; 95% CI 1.60–7.88, *P* = 0.002), and polymorphonuclear neutrophils (PMN; OR, 2.01; 95% CI 1.03–3.93, *P* = 0.041) (Table 2).

**Table 1:** Patient demographics

Characteristic	PJI (*N* = 87)	Non-PJI (*N* = 502)	*P*-value
Age	66.32 ± 10.13	62.28 ± 11.84	0.003
Sex			
Male	49 (56.3%)	282 (56.2%)	
Female	38 (43.7%)	220 (43.8%)	
BMI	22.99 ± 3.35	23.71 ± 3.25	0.047
Laterality			
Left	32 (36.8%)	235 (46.8%)	0.054
Right	55 (63.2%)	267 (53.2%)	
Risk factor			
Smoking	20 (23%)	124 (24.7%)	0.522
Alcohol	67 (77%)	378 (75.3%)	0.316
Laboratory markers			
CRP	43.66 ± 47.30	15.81 ± 28.80	< 0.001
ESR	44.38 ± 26.71	18.18 ± 17.29	< 0.001
WBC	8.15 ± 3.85	6.75 ± 2.05	< 0.001
PMN	0.67 ± 0.11	0.625 ± 0.10	< 0.001
PC	264.34 ± 112.43	236.48 ± 68.97	0.002
MPV	10.51 ± 1.06	10.69 ± 1.12	0.169
D-dimer	2.40 ± 3.21	1.10 ± 2.04	< 0.001
Investigation time	112.3 ± 24.2	98.7 ± 72.5	0.127
Comorbidities			
Diabetes	37 (42.5%)	151 (30.1%)	0.016
Hypertension	37 (42.5%)	193 (38.4%)	0.268

MPV, mean platelet volume

**Table 2:** Multivariate logistic regression analysis of independent risk factors for postoperative PJI in patients with THA

Variable	OR (95% CI)	*P*-value
CRP	5.03 (2.74–9.12)	< 0.001
ESR	3.65 (1.93–6.87)	< 0.001
PC	2.35 (1.17–4.73)	0.017
D-dimer	3.55 (1.60–7.88)	0.002
PMN	2.01 (1.03–3.93)	0.041

**Fig. 1:**
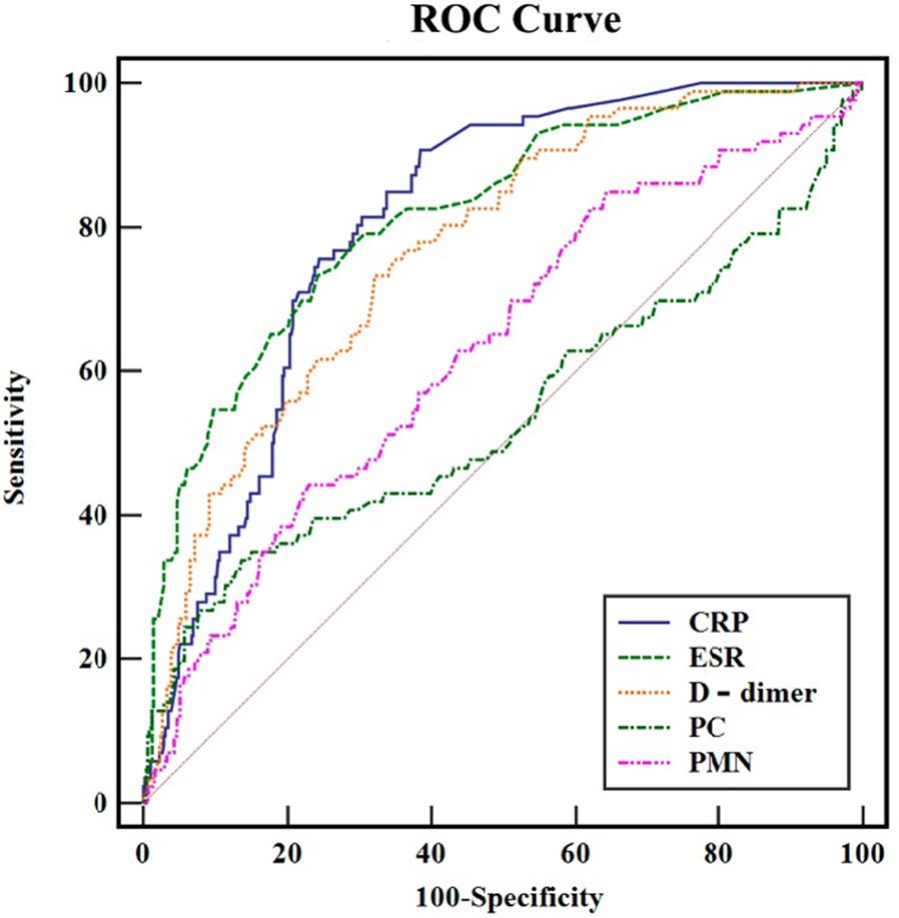
ROC curve of different detection markers for PJI diagnosis

ROC curve analysis evaluated the five independent risk factors’ area under curve (AUC) values, sensitivity, specificity, and optimal cutoff values (Table 3; Fig. 1). The ROC curve shows that ESR has the highest AUC (AUC, 0.816; 95% CI 0.782–0.847; *P* = 0.02), with a sensitivity of 78.16%, specificity of 69.32%, and the best cutoff point of > 20 mm/hr; CRP has the highest sensitivity (90.8%), an AUC of 0.805, and the best cutoff point of > 5.46 mg/L; and PC has the highest specificity (86.45%), an AUC of 0.534, and the best cutoff point of > 298 × 10^9^/L;

**Table 3:** Diagnostic value of different test markers for PJI patients

Variable	AUC	95% CI	Predictive cutoff	Sensitivity (%)	Specificity (%)
CRP	0.805	0.771–0.837	> 5.46 mg/L	90.80	61.43
D-dimer	0.770	0.734–0.804	> 0.94 μg/mL	73.56	67.33
ESR	0.816	0.782–0.847	> 20 mm/h	78.16	69.32
PC	0.534	0.492–0.575	> 298 × 10^9^/L	33.33	86.45
PMN	0.632	0.590–0.670	> 0.683	44.83	77.29

**Fig. 2:**
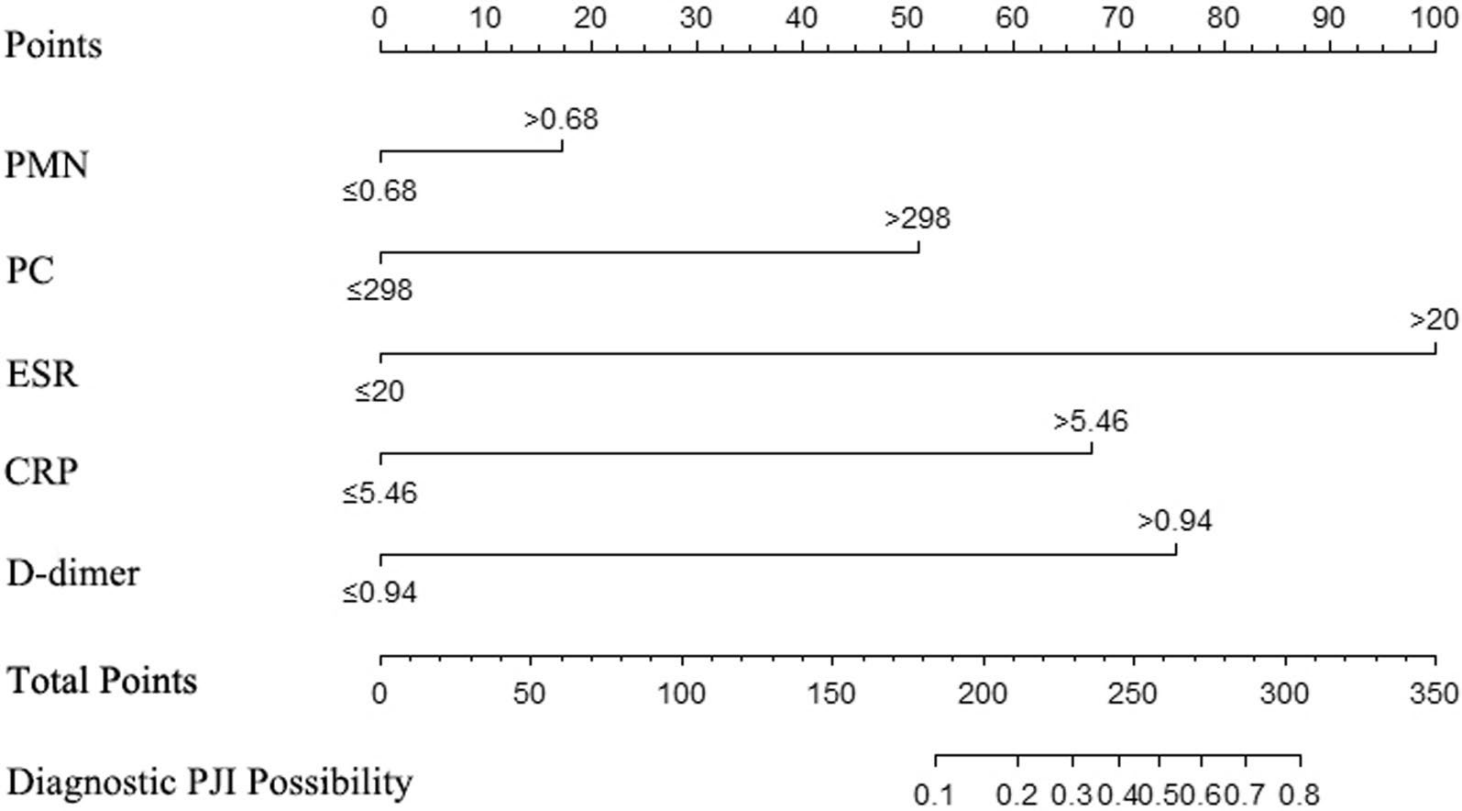
Establishing a nomogram for predicting PJI after THA

**Fig. 3:**
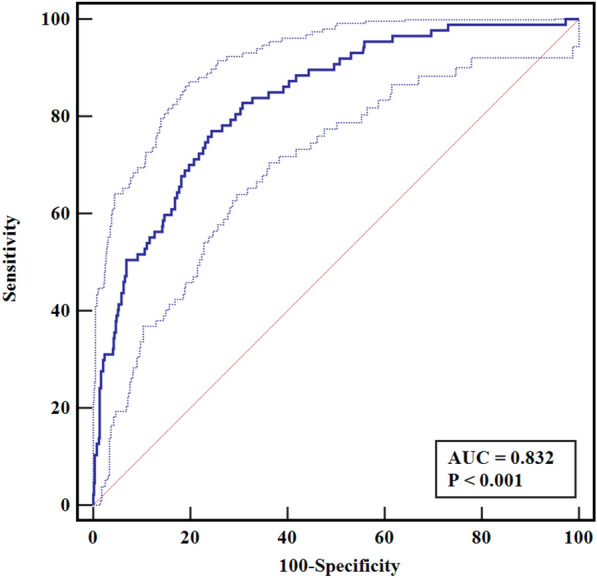
ROC curve was used to verify the discriminability of the nomogram prediction model

**Fig. 4:**
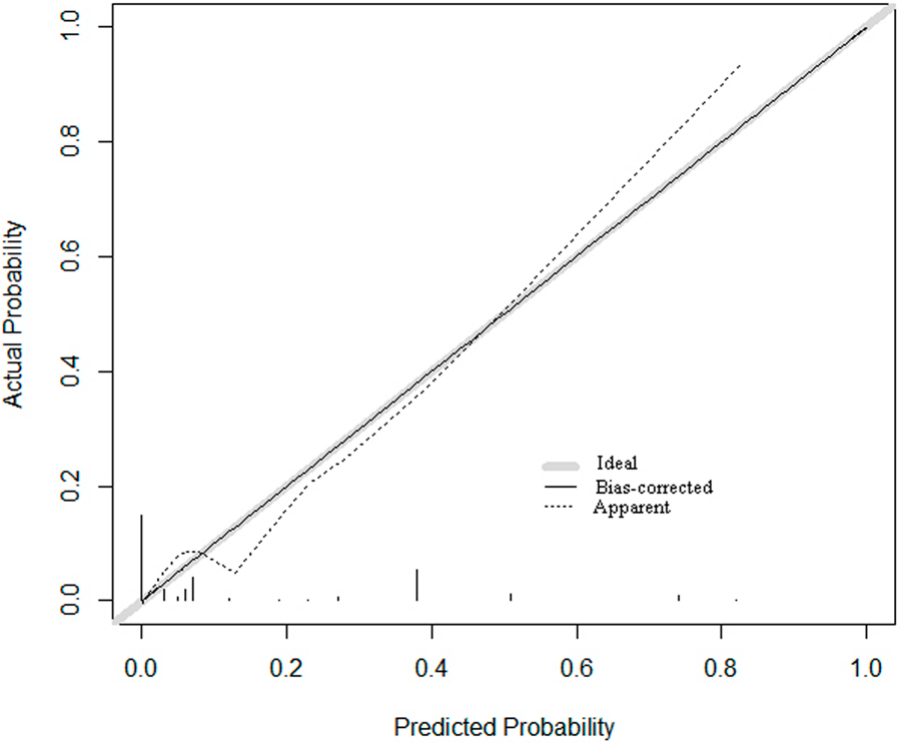
Nomogram calibration of postoperative prediction of PJI after THA

**Fig. 5:**
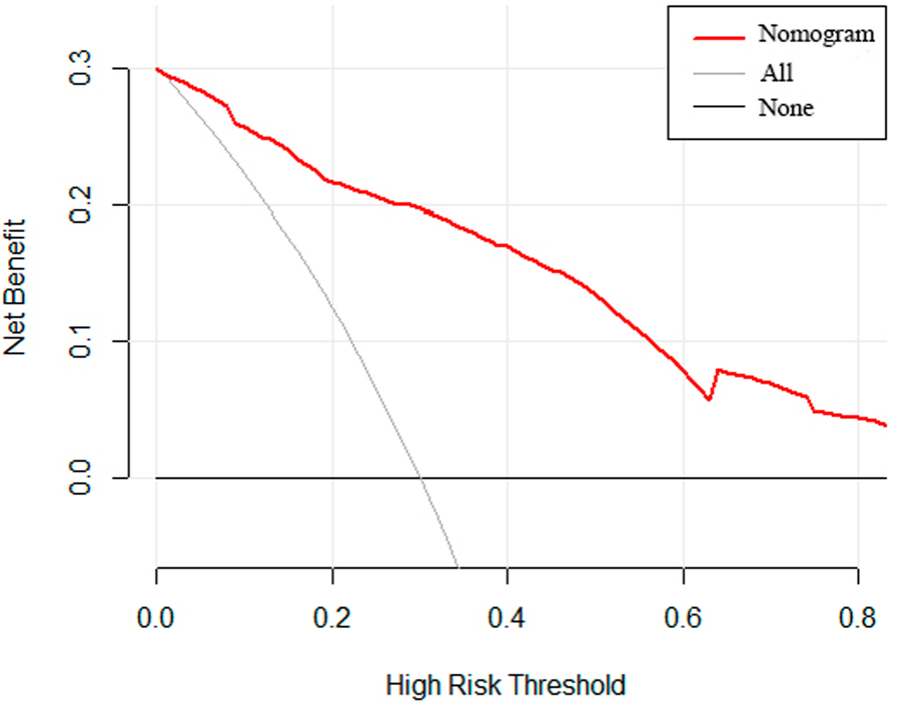
Clinical decision curve analysis of postoperative PJI in patients after THA

A nomogram was constructed on the basis of the five independent risk factors mentioned above (Fig. 2). In the nomogram, each independent risk factor is assigned a total score or a weighted total score (superscript), and the probability of PJI occurrence after post-THA is calculated according to the total score (subscript). The predictive performance of the model was assessed using the ROC curve (Fig. 3). The AUC of the nomogram was 0.832 (95% CI 0.798–0.861), with an optimal cutoff value of ≤ 0.883. At this cutoff, the Youden index was 0.5252, the sensitivity was 77.01%, and the specificity was 75.51%, indicating good discrimination by the nomogram. The Hosmer–Lemeshow test and calibration curve were used to evaluate the calibration of the model. The *P*-value of the Hosmer–Lemeshow test was 0.225, indicating good calibration. The calibration curve closely approximated the ideal 45° line, demonstrating a good agreement between the model’s predictions and observations (Fig. 4). Decision curve analysis (DCA) was used to evaluate the clinical utility of the nomogram. The DCA curve indicated that, when the threshold probability of PJI in patients with THA ranged from 0 to 0.8, the net benefit of using the nomogram was significantly higher than that of the “no intervention” and “all intervention” strategies (Fig. 5), suggesting that the model is clinically useful.

## Discussion

Our study attempts to fill this gap by determining and establishing the optimal thresholds for risk factors associated with PJI after hip arthroplasty and proposing a diagnostic nomogram for preliminary assessment. The nomogram includes CRP, ESR, PC, D-dimer, and PMN as predictive indicators for PJI following THA.

Previous studies have established that serum ESR and CRP are susceptible tests for diagnosing PJI [5, 8–10] and are practical screening tools. Multivariate logistic regression analysis and ROC curves have shown that CRP has the highest sensitivity, with the best cutoff point being 5.46 mg/L, close to the upper standard limit. One study reviewed a 1.2% reoperation rate within the first 6 weeks post-THA, with some cases presenting with PJI. It was found that serum CRP serves as an excellent screening test. However, the optimal threshold for diagnosing PJI in the acute postoperative phase is higher than the traditional values used for chronic PJI diagnosis [11]. Another study set the optimal cutoff value for diagnosing PJI after hip arthroplasty using CRP at 10 mg/L, with a sensitivity of 85.1% and a specificity of 67.6% [12]. These thresholds are significantly higher than those in our study. We believe there is a significant difference in CRP threshold values based on postoperative time thresholds [13]. The above studies only defined the postoperative time thresholds of 6 weeks and 4 weeks, whereas CRP levels gradually return to normal as the postoperative period extends [14]. According to the definitions by MSIS, PJI is classified into two types, acute and chronic, on the basis of a 90-day postoperative period, but this classification is currently controversial. This binary classification does not reflect the infection continuum and may not correspond to the infection’s pathophysiology or the pathogens’ behavior. Factors unique to each patient, such as immune status and comorbidities, can significantly influence the timing and presentation of PJI [15]. In this study, we excluded patients with systemic inflammatory diseases, because these can cause inflammatory reactions, which may lead to elevated laboratory indicators, thereby interfering with the accurate diagnosis of PJI.

Most PJIs are thought to be caused by low-virulence microorganisms, which may trigger a weak or absent CRP response [16]. These microorganisms preferentially adhere to the implant’s surface, forming a biofilm and evading the immune system, thus reducing the inflammatory response [17–19]. CRP, with its high sensitivity, is susceptible to influence from other infections and helps monitor the treatment of acute conditions rather than diagnosing PJI [20, 21]. To minimize false positives, it may be necessary to consider changes in CRP levels in PJI patients in the context of their overall health status. The longer half-life of ESR suggests it is more suitable for assessing chronic infections. Our study indicates that ESR has potentially higher diagnostic value, with the highest area under the curve, and warrants special attention in patients post-THA. Our findings are similar to those of Stephen P. Maier et al. [22], where ESR may remain elevated in chronic infections (ongoing active infection), while CRP is normal. Compared with No-PJI patients, PJI patients had higher PMN levels within the normal range, with a statistically significant difference. This is consistent with a study analyzing 1856 revision surgeries, which found the optimal threshold for percentage of PMN for sensitivity and specificity to be 69% [23]. In another study, when a cutoff of 90% was used for PMN, the summarized estimates of sensitivity, specificity, and LR for septic arthritis PMN were 60%, 78%, and 2.7 [24], respectively. However, our study’s best cutoff value for PMN only showed low sensitivity and moderate specificity. In our PJI cohort, PMN levels did not indicate systemic inflammation but gradually increased, suggesting PMN evaluation might be necessary, but its diagnostic cutoff remains contentious.

Systemic or local infections can alter coagulation and fibrinolytic activity, providing a new potential avenue for PJI diagnosis. During infection and inflammation, activated platelets can inhibit pathogen growth by activating immune cells and promoting their clearance [25]. Additionally, related research has confirmed that platelets become significant coordinators of inflammation and innate and adaptive immune responses through interactions with monocytes, neutrophils, lymphocytes, and endothelial cells [26]. Therefore, platelet count has emerged as a vital candidate indicator for PJI diagnosis in our study. Although the sensitivity of platelet count is not as high as CRP or ESR, it has shown the highest specificity (86.45%), indicating its potential contributory value in PJI diagnosis. In previous studies, serum D-dimer has been considered a promising biomarker for PJI, showing higher sensitivity and specificity than ESR and CRP. Still, our study did not confirm this finding. Although D-dimer is a risk factor, its sensitivity and specificity did not exceed those of CRP or ESR. In the research by Shahi et al. [27], racial differences in D-dimer levels between predominantly white (European American) and African American populations and our Asian cohort [28, 29] may account for this discrepancy. Therefore, the role of D-dimer as a diagnostic biomarker seems limited and should not replace established serum parameters.

Our study, through calibration analysis, has proven that traditional risk factor screening and a robust predictive nomogram are superior to univariate models. The DCA has verified the significant net benefit of the model, indicating its practical utility. According to the total score derived from Youden’s index, the probability of PJI is 0.128, with the highest sensitivity (82.76%) and specificity (70.12%). The nomogram suggests that performing joint fluid aspiration or culture is recommended for patients with a score greater than 200. For those with a score lower than 200, close monitoring and follow-up are advised, paying attention to the dynamic changes in serum markers and nomogram scores.

However, the study has several limitations. First, this is a retrospective study, and selection bias is inevitable. Secondly, due to the limited number of PJI patients, this preliminary prediction model requires multi-center external validation with a larger sample size. Third, as with most studies, we could not include all confounding factors or completely rule out the question of survivor bias. PJI often presents with systemic inflammatory diseases. By excluding these patients, our study sample may differ from the actual clinical population, thereby limiting the generalizability of our findings. This exclusion criterion means that our results may not be fully applicable to PJI patients with underlying autoimmune conditions. Future research should consider including patients with systemic inflammatory diseases to evaluate the predictive value of laboratory markers across a broader and more representative population.

In summary, the results of this study suggest that CRP, ESR, D-dimer, PMN, and PC may be independent risk factors for PJI after THA and have potential roles in the diagnosis of PJI. Furthermore, this study introduces a predictive model with good accuracy, which may assist clinicians in predicting the risk of PJI after THA, thereby facilitating timely medical interventions for both clinicians and patients. The effectiveness of the nomogram-based prediction model in early detection of PJI and improvement of patient outcomes warrants further investigation.

## Data Availability

The datasets used and/or analyzed during the current study are available from the corresponding author on reasonable request.

## Abbreviations

THA: Total hip arthroplasty
PJI: Periprosthetic joint infection
DCA: Decision curve analysis
ROC: Receiver operating characteristic curve
AUC: Area under curve
CRP: C-reactive protein
ESR: Erythrocyte sedimentation rate
PMN: Polymorphonuclear neutrophils
PC: Platelet count
TKA: Total knee arthroplasty
WBC: White blood cell count
MSIS: Musculoskeletal Infection Society
SD: Standard deviation
